# Elasticity evaluation of the plantar fascia: A shear wave elastography study involving 33 early-stage plantar fasciopathy subjects

**DOI:** 10.3389/fphys.2022.1060728

**Published:** 2022-12-16

**Authors:** Zhen-Zhen Jiang, Hua-Liang Shen, Qi Zhang, Gang Ye, Xiu-Cheng Li, Xia-Tian Liu

**Affiliations:** ^1^ Department of Ultrasound, Shaoxing People’s Hospital, Shaoxing, Zhejiang, China; ^2^ Pain Management, Shaoxing People’s Hospital, Shaoxing, Zhejiang, China; ^3^ Department of Orthopedics, Shaoxing People’s Hospital, Shaoxing, Zhejiang, China

**Keywords:** plantar fascia, plantar fasciitis, shear wave elastography, imaging modality, ultrasound

## Abstract

**Background:** Plantar fasciopathy, the most common foot condition seen in elderly and athletic populations, can be diagnosed and differentially diagnosed with imaging modalities such as ultrasound shear wave elastography (SWE). However, standard guidelines for ultrasound elastography of the plantar fascia are lacking. The purpose of this study was to determine the impact of the region of interest (ROI) on the evaluation of the plantar fascia elasticity and confirm the screening accuracy of SWE in the early-stage of plantar fasciopathy.

**Methods:** This was an observational case‒control study involving 50 feet of 33 early-stage plantar fasciopathy subjects (the plantar fasciopathy group) and 96 asymptomatic feet of 48 healthy volunteers (the non-pain group). Clinical information, including age, gender, height, weight, visual analogue scale (VAS) score, American Orthopaedic Foot and Ankle Scale score (AOFAS), and the symptom duration, were recorded. All participants underwent both conventional ultrasound and SWE evaluation. The plantar fascia elastic parameters included SWE_single-point_, calculated with a single-point ROI set at the greatest thickness of the plantar fascia, and SWE_multi-point,_ calculated by multipoint ROIs set continuously from the origin at the calcaneus to about 2 cm from the calcaneal origin.

**Results:** The plantar fasciopathy group presented a higher VAS score (median [IQR), 4.00 (3.00) vs. 0.00 (0.00), *p* < 0.001] and lower AOFAS score [median (IQR), 79.50 (3.00) vs. 100.00 (10.00), *p* < 0.001] than the non-pain group. The median plantar fascia thickness of the plantar fasciopathy group was significantly greater than that of the non-pain group [median (IQR), 3.95 (1.37) mm vs 2.40 (0.60) mm, *p* < 0.001]. Abnormal ultrasound features, including echogenicity, border irregularities, and blood flow signals, were more prominent in the plantar fasciopathy group than in the non-pain group (29% vs. 0%, *p* < 0.001; 26% vs. 1%, *p* < 0.001; 12% vs. 0%, *p* < 0.001, respectively). Quantitative analysis of the plantar fascia elasticity revealed that the difference between the value of SWE_single-point_ and SWE_multipoint_ was significant [median (IQR), 65.76 (58.58) vs. 57.42 (35.52) kPa, *p* = 0.02). There was a moderate and significant correlation between the value of SWE_single-point_ and heel pain. However, there was no correlation between the value of SWE_multipoint_ and heel pain. Finally, we utilized the results of SWE_single-point_ as the best elastic parameter reflecting clinical heel pain and found that SWE_single-point_ could provide additional value in screening early-stage plantar fasciopathy, with an increase in sensitivity from 76% to 92% over conventional ultrasound alone. Additionally, compared with conventional ultrasound and SWE, the use of both improved the accuracy of screening for plantar fasciopathy. Although there were no significant differences in the negative predictive value of conventional ultrasound, SWE, and their combination, the positive predictive value when using both (90.20%) was significantly greater than that when using conventional ultrasound (74.50%) or SWE alone (76.50%).

**Conclusion:** The plantar fascia elastic parameter calculated with single-point ROIs set at the greatest thickness of the plantar fascia is positively correlated with fascia feel pain. Single-point analysis is sufficient for the screening of the early-stage plantar fasciopathy using SWE. SWE_single-point_ may provide additional valuable information for assessing the severity of plantar fasciopathy.

## Introduction

Plantar heel pain, also known as “plantar fasciopathy”, is the most common foot condition seen in elderly and athletic populations (Monteagudo et al., 2018). It is estimated that approximately 2 million Americans suffer from plantar fasciopathy each year, corresponding to up to 10% of the population who experience plantar fasciopathy over the course of their lifetime (Martin et al., 2014). In both non-athletic and athletic populations, the prevalence of plantar fasciopathy significantly limits their physical activities and has a detrimental effect on health-related quality of life (Lin et al., 2022). Although imaging is not required for the diagnosis of plantar fasciopathy, it may help to rule out other alternate diagnoses of heel pain and establish the diagnosis if the termed diagnosis cannot be reached (Schneider et al., 2018). In addition, imaging modalities, such as ultrasound, have been reported to be suitable for guiding therapy procedures (Beydoğan and Yalçın, 2021).

Typical ultrasound features of plantar fasciopathy, such as plantar fascial thickening, fascial-border blurring, and hypoechoic echotextures, may not always be observed on conventional ultrasound in subjects with plantar heel pain (Sconfienza et al., 2013). Additionally, it has been reported that the thickness of the plantar fascia may not predict the functional outcome of plantar fasciopathy therapy (Ermutlu et al., 2018). Common therapies for plantar fasciopathy include conservative treatments (such as rest, ice, orthotics, physical stretching), corticosteroid injections, and even surgical treatments (Rasenberg et al., 2018; Wu FL. et al., 2019; Pinrattana et al., 2021; Rabadi et al., 2022). By either way, the basic principle of the treatment is to unload the stress over the plantar fascia. Consequently, the stress and stiffness evaluations of the plantar fascia may help to identify the therapy efficacy (Lin et al., 2015). Besides, differences in the percentage of softened plantar fascia have been observed in subjects with and without plantar fasciopathy (Lee et al., 2014). Shear wave elastography (SWE), a kind of ultrasound imaging modality that can quantitatively assess tissue stiffness using shear waves, has been reported to apply in the evaluation of plantar fascia. Table 1 summarizes the applications of SWE in the elasticity evaluation of the plantar fascia. A previous study placed the region of interest (ROI) for the SWE measurement in the area of the greatest plantar fascia thickness and revealed that SWE could distinguish symptomatic and asymptomatic subjects better than conventional ultrasound (Gatz et al., 2020). Another study set the ROI as the area including the calcaneal origin of the plantar fascia and 20 mm distal to the calcaneal origin for measuring the stiffness of the plantar fascia. The authors found that plantar fascia stiffness was not significantly different between males and females but was significantly lower in overweight subjects than in normal weight subjects (Taş et al., 2017). Additionally, one study defined the ROI by covering the boundaries of the plantar fascia to investigate the site and sex-differences in its mechanical properties (Shiotani et al., 2019). In these studies, the ROI differences in the stiffness of the plantar fascia stiffness were not investigated, which may affect the comparability of the studies. Additionally, the correlation between plantar fascia stiffness parameters and clinical parameters in early-stage plantar fasciopathy has not been confirmed.

**TABLE 1:** Applications of SWE in the elasticity evaluation of the plantar fascia.

Ref	Study purpose	No. Of the subjects	ROI setting	Results on the accuracy
Taş and Çetin, (2019)	To investigate the relationship between plantar pressure distribution and the mechanical properties of the plantar fascia and intrinsic foot muscles	41 healthy subjects	Multipoint measurements	NA
Shiotani, et al. (2019)	To investigate the site- and sex-differences in the mechanical properties of the plantar fascia	40 healthy subjects	Covering the boundaries of the plantar fascia	NA
Chino, et al. (2019)	To investigate the effect of toe dorsiflexion on the mechanical properties of the plantar fascia	16 healthy subjects	Covering the boundaries of the plantar fascia	NA
Gatz, et al. (2020)	To determine the value of SWE in evaluating plantar fascia	31 PF subjects and 10 healthy subjects	Single-point measurements	SWE: sensitivity (85%), specificity (83%), diagnostic accuracy (84%)
				SWE + B-mode: sensitivity (100%), specificity (81%), diagnostic accuracy (90%)
Beydoğan and Yalçın, (2021)	To evaluate the role of SWE in diagnosing plantar fascia	30 PF subjects and 40 healthy subjects	Three-point measurements	NA
Schillizzi, et al. (2021)	To compare elasticity features between symptomatic and asymptomatic subjects using SWE	19 PF subjects and 21 healthy subjects	Single-point measurements	NA
Current study	To determine the impact of the ROI on measurement of the plantar fascia stiffness and confirm the screening accuracy of SWE in the early-stage of plantar fasciopathy	33 PF subjects and 48 healthy subjects	Both Single-point and multipoint measurements	SWE: sensitivity (78%), specificity (87.5%), diagnostic accuracy (89%), PV (76.5%), NV (88.4%)
				SWE + conventional ultrasound: sensitivity (92%), specificity (85.42%), diagnostic accuracy (93.60%), PV (90.2%), NV (87.6%)

SWE, shear wave elastography; ROI, region of interest; PF, plantar fasciopathy; NA, not available; PV, positive predictive value; NV, negative predictive value.

Therefore, the purpose of our present study was to determine the impact of the ROI on measurement of the stiffness of the plantar fascia and confirm the screening accuracy of SWE in the early-stage of plantar fasciopathy. We took advantage of different SWE-measurement ROIs to comparatively analyse plantar fascia elasticity in asymptomatic and symptomatic participants and determine the correlation between different elastic parameters and clinical parameters. Furthermore, the sensitivity, specificity, and positive and negative predictive values of SWE, conventional ultrasound and their combination in the evaluation of the plantar fascia were determined.

## Materials and methods

### Study participants

Between January 2020 and June 2022, a total of 56 subjects who were evaluated for plantar fasciopathy by an orthopaedic specialist were screened in our study. The inclusion criteria were as follows: plantar heel tenderness without tenderness of other parts of the foot; and morning pain with the first few steps or worsening when weight bearing (Trojian and Tucker, 2019). The exclusion criteria were as follows: 1) Heel infection or the presence of a tumour; 2) systemic diseases, such as diabetes, rheumatoid arthritis, gout, mandatory spondylitis, *etc.*; 3) history of trauma or calcaneal fracture; 4) presence of calcaneal osteophytes as confirmed by X-ray examination; 5) history of previous surgery or any treatment for plantar fasciopathy in the past 3 months; and 6) history of pain for longer than 12 months.

**FIGURE 1:**
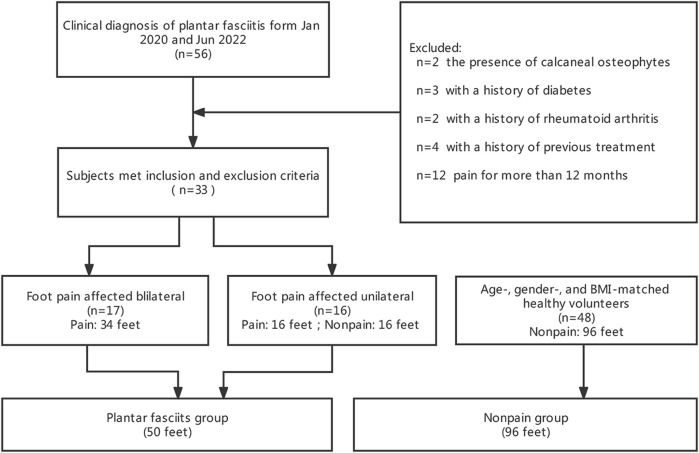
Enrollment flowchart of our study.

Based on the inclusion and exclusion criteria, 23 subjects were excluded, and 50 feet of 33 subjects, 17 of whom showed involvement of both feet, were classified as the plantar fasciopathy group and ultimately included in the analysis. As normal controls, 96 asymptomatic feet of 48 age-, gender, and body mass index (BMI)-matched healthy volunteers were classified as the non pain group (Figure 1). The study was approved by the Ethics Committee of Shaoxing People’s Hospital. All of the procedures were conducted according to the principles of the Declaration of Helsinki. Written informed consent was provided by all participants.

### Data collection

The clinical data of all participants, such as age, gender, height, weight, and the symptom durations were recorded. BMI was calculated as weight (kg)/squared value of height (m^2^). All participants underwent both conventional ultrasound and SWE evaluation. Furthermore, a visual analogue scale (VAS) (Schillizzi et al., 2021) and the American Orthopaedic Foot and Ankle Scale (AOFAS) (Kostuj et al., 2014) were used to evaluate the actual status of pain and foot function in the participants.

### Conventional ultrasound evaluation

To ensure consistency in the measurements and evaluation, both conventional and SWE ultrasound examinations were performed by a single trained and experienced sonographer who was blinded to the clinical findings of the participants using a Logiq E9 ultrasonic diagnostic system equipped with a linear 9 MHz transducer (GE Medical System, CA, United States). All the participants were prone positioned with their feet hanging over the edge of the examination bed. The transducer was placed over the surface of the heel, and ultrasound features of the plantar fascia, including thickness, echogenicity, border, and blood flow signals, were recorded. The thickness of the plantar fascia was measured at its thickest point of the anterior margin of the calcaneus in the longitudinal axes. Echogenicity abnormalities were defined as hypoechoic changes in the plantar fascia. All ultrasound images were stored for offline analyses.

### Shear wave elastography measurements

**FIGURE 2:**
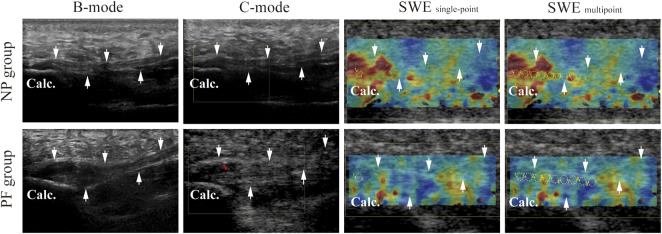
Acquisition plantar fascia images (arrows) of different ultrasound modalities in both NP group and PF group. Ultrasound modalities include B-mode, C-mode, and SWE. B-mode, and B-mode ultrasonography; C-mode, Color Doppler mode ultrasonography; SWE, shear wave elastography; NP group, non-pain group; PF group, plantar fasciopathy group; Calc, calcaneal.

Immediately after the conventional ultrasound examination, quantitative SWE measurements were performed. The transducer was carefully placed above the plantar fascia to avoid applying additional pressure. To avoid the tensile stress preloaded on the plantar fascia, the feet were maintained in a neutral position without any dorsiflexion of the ankle or toe. A circular ROI with a diameter of 2 mm was set as the measurement window, and quantitative elasticity values representing Young’s modulus were automatically calculated by the SWE system. To compare the difference between altered SWE measurements, both single-point analysis and multipoint analysis was conducted according to procedures modified from previous researches (Taş and Bek, 2018; Gatz et al., 2020). For single-point analysis, measurements were conducted in the area covering the greatest thickness of the plantar fascia in the longitudinal axes three times, and the means and standard deviations of the measurements were calculated. For multipoint analysis, measurements were conducted continuously from the origin at the calcaneus to approximately 2 cm from the calcaneal origin and then averaged to obtain the mean elasticity values of this region (Figure 2). The measurements were conducted three times. Means and standard deviations were calculated. The results of both SWE _single-point_ and SWE _multipoint_ were recorded for the subsequent analysis.

### Statistical analysis

Statistical analyses were conducted with SPSS version 26.0 (SPSS Inc., Chicago, IL) and MedCalc version 15.2.2 (MedCalc Inc., Mariakerke, Belgium). The Kolmogorov–Smirnov test was performed to determine whether the data were normally distributed. The results revealed that data of BMI, plantar fascia thickness, and value of SWE _single-point_ in the plantar fasciopathy group were normal distributed, while the remaining continuous variables were all non-normally distributed. Therefore, the data were presented as medians (interquartile ranges, IQR), while categorial variables were presented as percentages. Group differences for continuous variables were examined by using the Mann‒Whitney *U* test. Paired statistical analyses were performed with the Wilcoxon signed-rank test. The chi-square test was conducted to identify the differences in imaging characteristics between groups. Spearman’s correlation test was used to assess the correlation between the clinical data and ultrasound parameters. Receiver operating characteristic (ROC) curve analysis, including calculation of the area under the curve (AUC) was performed to identify the screening performance of the different ultrasound modalities. *p* < 0.05 was considered statistically significant (Gatz et al., 2020).

G*Power 3.1 (Düsseldorf, Germany) was used to calculate the required number of subjects. According to the results of a previous pilot study (Aggarwal et al., 2020) about the evaluation of plantar fascia using ultrasonography in individuals with heel pain and normal volunteers, to achieve an *α* level of 0.05, and a power level of 0.85, the calculated effect size was about 0.63. To evaluate the plantar fascia using SWE in individuals with heel pain and normal volunteers, we set the expected effect size as reported, that is, 0.63. Then an *α* level as 0.05, and a power level of 0.90 were set. After the calculation of G*Power, a sample size of 142 subjects (47 for the experimental group and 95 for the control group) would be required to present significant differences in the comparisons of the two groups.

## Results

### Clinical characteristics of the participants

The demographic data of the participants, including age, gender, weight, height, BMI, VAS score, AOFAS score, and the symptom durations are summarized in Table 2. The median age of the participants was 56 years (IQR: 25.25 years), and 88 (60.30%) participants were female. Although participants in the plantar fasciopathy group had a higher weight and height [median (IQR), 67.50 (13.00) vs. 60.00 (17.50) kg, *p* = 0.006, and 1.67 (0.14) vs. 1.63 (0.11) m, *p* = 0.012, respectively] than those in the non-pain group, the differences were not significant for age, gender or BMI (all *p* > 0.05). Participants in the plantar fasciopathy group had significantly higher VAS scores [median (IQR), 4.00 (3.00) vs. 0.00 (1.00), *p* < 0.001] and significantly lower AOFAS scores [median (IQR), 79.50 (3.00) vs.100.00 (10.00), *p* < 0.001] than those in the non-pain group. The median symptom duration in the plantar fasciopathy group was 6 months (IQR: 3 months). In our study, there were no acute plantar fasciopathy participants whose symptom duration were less than 1 month.

**TABLE 2:** Baseline characteristics of the subjects.

	Overall (*n* = 146)	Nonpain group (*n* = 96)	Plantar fasciopathyGroup (*n* = 50)	p-value
Age (years)	56.00 (25.25)	53.00 (29.75)	57.50 (15.25)	0.606
Female (n, %)	88 (60.30)	62 (64.60)	26 (52.00)	0.157
Weight (kg)	60.00 (17.25)	60.00 (17.50)	67.00 (13.00)	**0.012**
Height (m)	1.63 (0.12)	1.63 (0.11)	1.67 (0.14)	**0.032**
BMI (kg/m2)	23.39 (4.38)	22.59 (4.87)	24.06 (2.80)	0.083
VAS score (point)	0.50 (3.00)	0.00 (0.00)	4.00 (3.00)	**< 0.001**
AOFAS score (point)	90.00 (20.00)	100.00 (10.00)	79.50 (3.00)	**< 0.001**
Symptom duration (months)	0 (4)	6 (3)	0 (0)	**< 0.001**

Data are presented as number (%) or median (Interquartile range). Bold font indicates a significant correlation (*p* < 0.05). BMI, body mass index; VAS, visual analogue scale; AOFAS, American Orthopedic Foot and Ankle Society score.

### Ultrasound features of the participants

The ultrasound features of the participants are listed in Table 3. The median plantar fascia thickness of the plantar fasciopathy group, 3.95 mm (IQR: 1.37 mm), was significantly greater than that of the non-pain group (2.40 mm, IQR: 0.60 mm). Regarding abnormal ultrasound features of the plantar fascia among the participants, 29% had hypoechoic changes, 26% showed border irregularities, and 12% demonstrated blood flow signals. All three of these abnormal features appeared more prominently in the plantar fasciopathy group than in the non-pain group (29% vs. 0%, *p* < 0.001; 26% vs. 1%, *p* < 0.001; 12% vs. 0%, *p* < 0.001, respectively).

**TABLE 3:** Ultrasound features of the subjects in different groups.

	Overall (*n* = 146)	Nonpain group (*n* = 96)	Plantar fasciopathy group (*n* = 50)	*p*-value
Thickness of plantar fascia (mm)	2.65 (1.40)	2.40 (0.60)	3.95 (1.37)	**< 0.001**
Hypoechogenicity (n, %)	29 (19.90)	0 (0.00)	29 (58.00)	**< 0.001**
Border irregularities (n, %)	27 (18.50)	1 (1.00)	26 (52.00)	**< 0.001**
Blood flow signals (n, %)	12 (8.20)	0 (0.00)	12 (24.00)	**< 0.001**
SWE _single-point_ (kPa)	65.76 (58.58)	85.63 (49.53)	34.98 (25.46)	**< 0.001**
SWE _multipoint_ (kPa)	57.42 (35.52)*	58.14 (34.00)*	51.13 (36.91)*	**0.045**

Data are presented as number (%) or median (Interquartile range).

*Compared with SWE _one-point_ (*p* < 0.05). Bold font indicates a significant correlation (*p* < 0.05). SWE, shear wave elastography.

Quantitative analysis of the plantar fascia elasticity revealed that in both the single-point and multipoint analyses, the SWE values of the non-pain group were significantly higher than those of the plantar fasciopathy group [median (IQR), 85.63 (49.53) vs. 34.98 (25.46) kPa, *p* < 0.001, 58.14 (34.00) vs. 51.13 (36.91) kPa, *p* = 0.045, respectively]. There was a significant difference between the SWE value of the single-point analysis and multipoint analysis [median (IQR), 65.76 (58.58) vs. 57.42 (35.52) kPa, *p* = 0.02]. In the non-pain group, the SWE value of the single-point analysis was higher than that of the multipoint analysis [median (IQR), 85.63 (49.53) vs. 58.14 (34.00) kPa, *p* < 0.001]. In the plantar fasciopathy group, the SWE value of the single-point analysis was lower than that of the multipoint analysis [median (IQR), 34.98 (25.46) vs. 51.13 (36.91) kPa, *p* < 0.001].

### Correlations between the ultrasound parameters and clinical parameters

Table 4 shows the correlations between the ultrasound parameters and clinical parameters. The thickness of the plantar fascia as measured by conventional ultrasound was positively correlated with age (*r* = 0.213, *p* = 0.01), and BMI (*r* = 0.284, *p* = 0.001), whereas the plantar fascia thickness was negatively correlated with AOFAS score (*r* = −0.374, *p* < 0.001), but strongly positively correlated with the symptom duration (*r* = 0.586, *p* < 0.001). The value of SWE _single-point_ was strongly positively correlated with AOFAS score (*r* = 0.521, *p* < 0.001), whereas negatively correlated with VAS score (*r* = −0.604, *p* < 0.001) and the symptom duration (r = −0.618, *p* < 0.001). The value of SWE _multipoint_ was not correlated with BMI (*r* = 0.007, *p* = 0.935), VAS score (*r* = −0.120, *p* = 0.138), or AOFAS score (*r* = 0.053, *p* = 0.522), whereas was mildly correlated with age (*r* = 0.242, *p* = 0.003) and the symptom duration (*r* = −0.182, *p* = 0.028).

**TABLE 4:** Correlations among ultrasound parameters and clinical parameters.

		Age	BMI	VAS score	AOFAS score	Symptom duration	Thickness of plantar fascia	SWE _single-point_	SWE _multipoint_
Age	*r*	—	**0.327**	**0.174**	**−0.338**	0.046	**0.213**	0.056	**0.242**
	*P*	—	**< 0.001**	**0.035**	**< 0.001**	0.582	**0.010**	0.502	**0.003**
BMI	*r*	**0.327**	—	0.101	**−**0.148	0.156	**0.284**	**−**0.066	0.007
	*P*	**< 0.001**	—	0.224	0.075	0.061	**0.001**	0.427	0.935
VAS score	*r*	**0.174**	—	—	**−0.933**	**0.853**	**0.529**	**−0.604**	**−**0.120
	*P*	**0.035**	—	—	**< 0.001**	**< 0.001**	**< 0.001**	**< 0.001**	0.148
AOFAS score	*R*	**−0.338**	—	**- 0.933**	—	**−0.820**	**−0.577**	**0.521**	0.053
	*P*	**< 0.001**	—	**< 0.001**	—	**< 0.001**	**< 0.001**	**< 0.001**	0.522
Symptom duration	*r*	—	—	**0.853**	**−0.820**	—	**0.586**	**−0.618**	**−0.182**
	*P*	—	—	**< 0.001**	**< 0.001**	—	**< 0.001**	**< 0.001**	**0.028**
Thickness of plantar fascia	*r*	**0.213**	**0.284**	**0.529**	**−0.577**	**0.586**	—	**−0.374**	**−**0.123
	*P*	**0.010**	**0.001**	**< 0.001**	**< 0.001**	**< 0.001**	—	**< 0.001**	0.138
SWE _single-point_	*r*	—	—	**−0.604**	**0.521**	**−0.618**	**−0.374**		**0.385**
	*P*	—	—	**< 0.001**	**< 0.001**	**< 0.001**	**< 0.001**		**< 0.001**
SWE _multipoint_	*r*	**0.242**	—	—	—	**−0.182**	—	**0.385**	—
	*P*	**0.003**	—	—	—	**0.028**	—	**0.000**	—

The upper-right part of the table shows all correlations (*r* = CC, and P) among ultrasound parameters and clinical parameters in the subjects. The lower-center panel represents significant correlations (*p* < 0.05). Bold font indicates a significant correlation (*p* < 0.05). BMI, body mass index; VAS, visual analogue scale; AOFAS, american orthopedic foot and ankle society score; SWE, shear wave elastography; r, correlation coefficient; P, significance.

### Performance of different evaluation methods in the plantar fasciopathy screening

**FIGURE 3:**
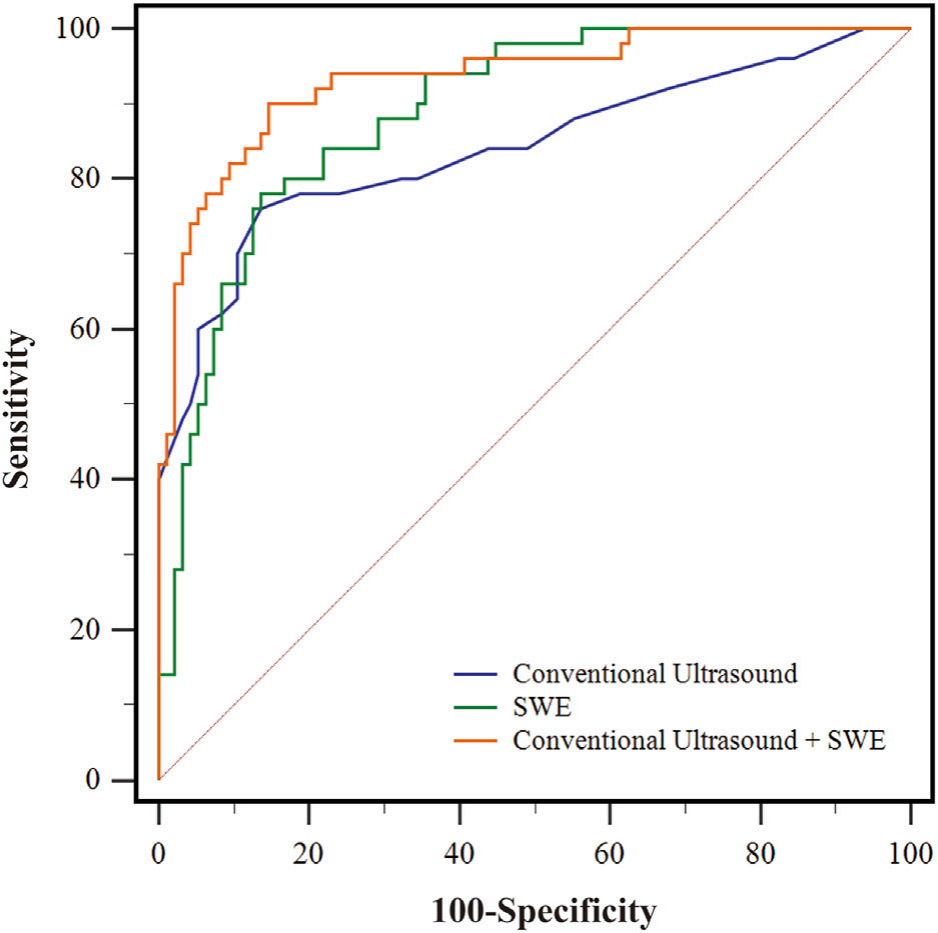
The receiver operating characteristic (ROC) curves of different evaluation method for screening early-stage plantar fasciopathy. The blue line represents the ROC curve of conventional ultrasound. The green line represents the ROC curve of SWE. The orange line represents the ROC curve of conventional ultrasound + SWE.

The screening efficacy of conventional ultrasound, SWE, and their combination were calculated. As shown in Table 5, conventional ultrasound alone had the lowest sensitivity in screening plantar fasciopathy, at 76%, and a specificity of 86.46%, while SWE alone was slightly more sensitive (78%) and specific (87.50%). Nevertheless, the combination of conventional ultrasound and SWE increased the sensitivity to 92% but reduced the specificity to 85.42%. The AUC of conventional ultrasound + SWE was higher than that of conventional ultrasound [0.936 (0.883, 0.970) vs. 0.846 (0.777, 0.901), *p* < 0.001] and SWE alone [0.936 (0.883, 0.970) vs. 0.890 (0.827, 0.935), *p* = 0.027], while no significant difference was found between the individual imaging modalities [conventional ultrasound, 0.846 (0.777, 0.901) vs. SWE, 0.890 (0.827, 0.935), *p* = 0.304) (Figure 3). The positive predictive value of conventional ultrasound + SWE (90.20%) was significantly higher than that of conventional ultrasound (74.50%) and SWE alone (76.50%), although no evidence of a difference was found in the negative predictive value among the three methods (conventional ultrasound: 87.40%; SWE: 88.40%; conventional ultrasound + SWE: 87.60%) (Table 5).

**TABLE 5:** Comparison of the diagnostic value of Conventional ultrasound, SWE, and Conventional ultrasound + SWE.

Statistics	Conventional ultrasound	SWE	Conventional ultrasound + SWE
AUC (95% CI)	0.846 (0.777, 0.901)*	0.890 (0.827, 0.935)*	0.936 (0.883, 0.970)
Sensitivity (95% CI, %)	76.00 (61.80, 86.90)	78.00 (64.00, 88.50)	92.00 (80.80, 97.80)
Specificity (95% CI, %)	86.46 (78.00, 92.60)	87.50 (79.20, 93.40)	85.42 (76.70, 91.80)
Positive predictive value (95% CI, %)	74.50 (63.30, 83.20)	76.50 (65.20, 84.90)	90.20 (77.80, 96.10)
Negative predictive value (95% CI, %)	87.40 (80.80, 91.90)*	88.40 (81.80, 92.80)*	87.60 (81.60, 91.90)

*Compared with Conventional ultrasound + SWE (*p* < 0.05).

AUC, area under curve; CI, confidence interval; SWE, shear wave elastography.

## Discussion

In the present study, we evaluated the correlation between different elastic parameters and clinical parameters and revealed that the plantar fascia elastic parameters calculated by single-point analysis were moderately and significantly correlated with foot pain as evaluated by the VAS score and AOFAS score. Our study is the first to discuss the impact of different ROI selections in evaluating the elasticity properties of the plantar fascia. Single-point analysis was sufficient for the evaluation of the plantar fascia using SWE, which has not been reported by previous studies. Furthermore, the screening value of SWE using single-point analysis for the identification of early-stage plantar fasciopathy was confirmed.

Plantar fasciopathy is the most common foot condition encountered by clinicians. It was reported that 80% of subjects with plantar fasciopathy could have symptom resolutions within 12 months with proper non-operative therapies (Trojian and Tucker, 2019). The subjects whose symptom duration longer than 1 year usually indicate a chronic condition (Martin et al., 2014). Hence, the subjects whose symptom duration less than 12 months were classified as the early-stage plantar fasciopathy in our study. To confirm the screening accuracy of SWE in early-stage plantar fasciopathy, the subjects whose symptom duration longer than 12 months were excluded. Besides, the median symptom duration in the plantar fasciopathy group in our study was 6 months (IQR: 3 months). There were no acute plantar fasciitis subjects (symptom duration less than 1 month) in our study. We assume the reason may be that during the acute stage of plantar fasciopathy, the patients usually do not visit hospitals.

Plantar fasciopathy needs to be differentiated from other causes of plantar heel pain, such as calcaneal osteophytes, calcaneal fracture, heel fat pad atrophy, the existence of cysts or tumours, and tarsal tunnel syndrome, and with the help of imaging findings (Tu, 2018). As one of the most common and readily available imaging modalities routinely used in the clinic, ultrasound has been proven useful in measuring the plantar fascia thickness (Karabay et al., 2007). In our study, the median thickness of the plantar fascia in the plantar fasciopathy group was significantly greater than that in the non-pain group [3.95 (1.37) mm vs. 2.40 (0.60) mm, *p* < 0.001]. Abnormal ultrasound features, including echogenicity, border irregularities, and blood flow signals, were more prominent in the plantar fasciopathy group than in the non-pain group (29% vs. 0%, *p* < 0.001; 26% vs. 1%, *p* < 0.001; 12% vs. 0%, *p* < 0.001, respectively), which is consistent with previous studies (Wu J. et al., 2019; Aggarwal et al., 2020). Although the thickness of the plantar fascia was reported to be associated with soft tissue stiffness (Lin et al., 2015), we only found a correlation between the plantar fascia thickness and the value of SWE _single-point_ but not between the plantar fascia thickness and the value of SWE _multipoint_. We speculated that the average plantar fascia elasticity obtained by multipoint analysis failed to reflect the elasticity of the thickest region of the plantar fascia, so no correlations were found between the plantar fascia thickness and the value of the SWE _multipoint_. Although the thickness of the plantar fascia measured on ultrasound was correlated with the clinical scores as well as with age and BMI in our study, the association between changes in plantar fascia thickness and the degree of plantar fasciopathy is controversial (Maki et al., 2017; Gamba et al., 2018). Elastography could be used as a screening tool to indicate early changes in the stiffness of the plantar fascia in symptomatic patients if the conventional ultrasound findings are insufficient (Gatz et al., 2020). However, there is currently a lack of standard guidelines for ultrasound elastography of the plantar fascia. Some factors may influence the elasticity value of the plantar fascia, including the intensity of walking, age, sex, somatotype, and compressive force (Taş et al., 2017; Shiotani et al., 2019; Lung et al., 2020). In addition, previous studies used different methods to evaluate the elasticity of the plantar fascia, affecting the comparability of these results (Putz et al., 2017; Taş and Bek, 2018). To exclude the influence of confounders, a total of 50 feet from a plantar fasciopathy population and 96 feet from an age-, gender, and BMI-matched asymptomatic population were included to compare the results from two different image acquisition ROIs. As shown in Table 2, the VAS score and AOFAS score were higher in the plantar fasciopathy group than in the non-pain group, which was consistent with the corresponding clinical symptoms. Then, the correlation between the elastic parameters of the plantar fascia and foot pain was analysed.

In terms of the elasticity evaluation of plantar fascia, initially semi-quantitative analyses were conducted. By taking advantage of real-time ultrasound elastography and a visual grading system, previous researchers found that the plantar fascia was softened in plantar fasciopathy subjects (Wu et al., 2011; Sconfienza et al., 2013; Lee et al., 2014). However, the elastography methods in these studies required an operator and failed to represent the tissue stiffness quantitatively (Ríos-Díaz et al., 2015). To achieve more objective outcomes, SWE, which employs an acoustic push pulse, was introduced for the measurement of the intrinsic elasticity of the plantar fascia. Due to the use of non-manual compression techniques, SWE is reproducible and has little operator dependence (Gangadhar et al., 2016). However, there are several factors that may affect the outcomes of SWE in the evaluation of plantar fascia. Chino et al. (2019) studied the effect of toe dorsiflexion on the shear wave speed in different regions of the plantar fascia and revealed that it could induce inhomogeneous tensile stress within the tissue. When the toe was dorsiflexed, increased shear wave speed was detected in the distal region, while no differences were observed in the region of origin. They assumed it was the larger cross-sectional area of the plantar fascia in the original region that made the tensile stress less sensitive to changes in tensile force. Additionally, other studies on plantar fascia elastography have focused on the screening accuracy, intra- and interobserver agreements, and other influencing factors (Gatz et al., 2020; Beydoğan and Yalçın, 2021; Lin et al., 2022).

Nevertheless, none of these studies discussed the variability of elasticity values across different selections of ROIs for stiffness analysis. Since there are no specific guidelines for image acquisition regarding calculating the mean stiffness of the plantar fascia, it appears that our study is the first to discuss the impact of different ROI selections in evaluating the elasticity properties of the plantar fascia. Our results revealed that in both the single-point and multipoint analyses, the SWE stiffness values of the non-pain group were significantly higher than those of the plantar fasciopathy group [85.63 (49.53) vs. 34.98 (25.46) kPa, *p* < 0.001, 58.14 (34.00) vs. 51.13 (36.91) kPa, *p* = 0.045, respectively], indicating that the plantar fasciae in the non-pain group were “harder” or “more elastic” than those in the plantar fasciopathy group, which is in line with previous studies (Gatz et al., 2020; Schillizzi et al., 2021). It should also be noted that the difference between the SWE values from single-point analysis and multipoint analysis was significant [65.76 (58.58) vs. 57.42 (35.52) kPa, *p* = 0.02]. In the non-pain group, the value of SWE _single-point_ was higher than that of SWE _multipoint_ [85.63 (49.53) vs. 58.14 (34.00) kPa, *p* < 0.001], while in the plantar fasciopathy group, the value of SWE _single-point_ was lower than that of SWE _multipoint_ [34.98 (25.46) vs. 51.13 (36.91) kPa, *p* < 0.001]. This disparity may be attributable to the different levels of tensile stress experienced by different regions of the plantar fascia, which result in distinct histological changes in the tissue (Ballal et al., 2014). Our study also revealed that the value of SWE _single-point_ was strongly correlated with clinical parameters such as AOFAS score, VAS score and the symptom duration, whereas the value of SWE _multipoint_ was not correlated or only was mildly correlated to clinical parameters, which indicated that multiple point measurements were less sensitive in reflecting the clinical symptoms of fascia heel pain than single-point analysis. The reason may be due to the wash-out effect. Averaging some less significant points might wash out the influence of significant points. It was reported that at the greatest thickness of the plantar fascia in plantar fasciopathy patients, the degenerative processes cause collagen breakdown, fibroblast hypertrophy, and matrix degradation, which finally leads to softening of the plantar fascia (Schillizzi et al., 2021). From this point of view, evaluating the elasticity at the greatest thickness of the plantar fascia could better reflects the pathological state of plantar fasciopathy, which was consistent with our findings.

Finally, we utilized the results of SWE _single-point_ as the best elastic parameter reflecting the degree of clinical heel pain and found that provides additional value in screening early-stage plantar fasciopathy, increasing the sensitivity from 76% to 92% over conventional ultrasound alone. Additionally, compared with conventional ultrasound (AUC 0.846) and SWE (AUC 0.890), the use of both (AUC 0.936) improved the accuracy of screening for early-stage plantar fasciopathy. Although there were no significant differences in the negative predictive value of conventional ultrasound, SWE, and their combination, the positive predictive value of using both (90.20%) increased significantly from using conventional ultrasound (74.50%) or SWE alone (76.50%). Table 1 summarized the results on the accuracy of SWE in evaluating the plantar fascia in previous studies. Compared to our results, Gatz et al. (2020) reported a higher sensitivity for the combination of SWE and conventional ultrasound (100%) in screening plantar fasciopathy, which may be due to the differences between the study populations. In their study, the authors included subjects with a longer disease history, which may have caused the greater imaging positive rate of the study population. In contrast, our study only evaluated subjects with heel pain for no longer than 12 months. Our present results showed that greyscale ultrasonography in combination with the value of SWE _single-point_ could be used to assess not only the morphology but also the stiffness of the plantar fascia, which may provide additional valuable information for assessing the severity of plantar fasciopathy and be used as guidance for developing therapeutic regimens.

## Limitations

There are several limitations in the current study. First, rather than manually drawn ROIs, fixed-size circular ROIs were set as the measurement windows for the quantitative analysis in our study. Although this is a more reliable measurement method in terms of minimizing researcher bias, the results may not represent the entire stiffness of the plantar fascia. However, it was previously reported that in more than two-thirds of patients, plantar fasciopathy occurs in the proximal fascia (Ieong et al., 2013). In addition, the aim of our study was to confirm the best elastic parameter reflecting clinical heel pain. According to our analysis, foot paint was best reflected by the elasticity value of the greatest thickness, rather than the average elasticity, of the plantar fascia. By acquiring multiple measurements and taking the average values within the fixed-size ROIs, SWE was sufficiently robust for plantar fasciopathy screening. Second, this was an observational case‒control study, and selection bias may exist. Third, the influence of foot type was not covered in our study. It was reported that the foot type would influence the intrinsic muscles and plantar fascia (Chuckpaiwong et al., 2008). Further study may focus on the effect of foot type on the plantar elasticity evaluation. Finally, all image acquisitions were performed by a single trained and experienced sonographer. Although this design may ensure consistency in the measurements, evaluations of intra- and interobserver agreements were impossible. However, as a non-manual compression technique, SWE has been reported to have little operator dependence and to be highly reproducible. To further validate the reliability of SWE in the evaluation of plantar fascia, evaluations of intra- and interobserver agreements could be conducted.

## Conclusion

In conclusion, the plantar fascia elastic parameter calculated by a single-point ROI set at the greatest thickness of the plantar fascia, rather than a multipoint ROI spanning from the origin at the calcaneus to approximately 2 cm from the calcaneal origin, was positively correlated with fascia heel pain. Single-point analysis was thus sufficient for the evaluation of the plantar fascia using SWE. Additionally, SWE _single-point_ may provide additional value in screening early-stage plantar fasciopathy, increasing the sensitivity from 76% to 92% when combined with conventional ultrasound. SWE _single-point_ may provide additional valuable information for assessing the severity of plantar fasciopathy and may be used as guidance for developing therapeutic regimens.

## Data Availability

The raw data supporting the conclusion of this article will be made available by the authors, without undue reservation.
